# Potential for the Australian and New Zealand paediatric intensive care registry to enhance acute flaccid paralysis surveillance in Australia: a data-linkage study

**DOI:** 10.1186/1471-2334-13-384

**Published:** 2013-08-21

**Authors:** Linda K Hobday, Bruce R Thorley, Janet Alexander, Thomas Aitken, Peter D Massey, Michelle Cretikos, Anthony Slater, David N Durrheim

**Affiliations:** 1 National Enterovirus Reference Laboratory, Victorian Infectious Diseases Reference Laboratory, North Melbourne, Victoria, Australia; 2 Australian and New Zealand Intensive Care Society, Herston, Queensland, Australia; 3 Hunter New England Population Health, Wallsend, NSW, Australia; 4 School of Health, University of New England, Armidale, NSW, Australia; 5 School of Public Health, University of Sydney, Darlington, NSW, Australia; 6 Centre for Epidemiology and Evidence, NSW Ministry of Health, Sydney, NSW, Australia; 7 Paediatric Intensive Care Unit, Royal Children’s Hospital, Herston, Queensland, Australia; 8 Hunter Medical Research Institute, New Lambton, NSW, Australia

**Keywords:** Acute flaccid paralysis, Clinical surveillance, Poliovirus, Paediatric intensive care

## Abstract

**Background:**

Australia uses acute flaccid paralysis (AFP) surveillance to monitor its polio-free status. The World Health Organization criterion for a sensitive AFP surveillance system is the annual detection of at least one non-polio AFP case per 100,000 children aged less than 15 years, a target Australia has not consistently achieved. Children exhibiting AFP are likely to be hospitalised and may be admitted to an intensive care unit. This provides a potential opportunity for active AFP surveillance.

**Methods:**

A data-linkage study for the period from 1 January 2005 to 31 December 2008 compared 165 non-polio AFP cases classified by the Polio Expert Panel with 880 acute neurological presentations potentially compatible with AFP documented in the Australian and New Zealand Paediatric Intensive Care (ANZPIC) Registry.

**Results:**

Forty-two (25%) AFP cases classified by the Polio Expert Panel were matched to case records in the ANZPIC Registry. Of these, nineteen (45%) cases were classified as Guillain-Barré syndrome on both registries. Ten additional Guillain-Barré syndrome cases recorded in the ANZPIC Registry were not notified to the national AFP surveillance system.

**Conclusions:**

The identification of a further ten AFP cases supports inclusion of intensive care units in national AFP surveillance, particularly specialist paediatric intensive care units, to identify AFP cases that may not otherwise be reported to the national surveillance system.

## Background

The Global Polio Eradication Initiative has made tremendous progress towards the goal of eradicating polio worldwide. In 1988, at the time of the initiative’s inception, there were more than 350,000 polio cases reported from 125 countries [1]. In 2013, only three countries remain endemic: Afghanistan, Nigeria and Pakistan. India was removed from the list of endemic countries in January 2012 after remaining polio-free for one year, which was a monumental achievement for the polio eradication program [2]. The World Health Organization (WHO) Western Pacific region, which includes Australia, was certified polio-free in 2000 [3]. However, the region remains vulnerable to importation of wild poliovirus, as occurred in China during 2011 [4] and Australia in July 2007, with disease confirmed in a 22 year old student returning from Pakistan [5]. Australia has used inactivated polio vaccine exclusively since November 2005, which is funded under the National Immunisation Program (NIP) for children. The WHO recommends that all polio non-endemic countries conduct acute flaccid paralysis (AFP) surveillance in children less than 15 years of age to monitor and verify polio-free status. The WHO has defined a non-polio background AFP surveillance rate that serves to confirm the sensitivity of a non-endemic country’s polio surveillance. To maintain WHO certification as polio-free, Australia is expected to:

1) detect at least one case of non-polio AFP for every 100,000 children less than 15 years of age annually;

2) collect two faecal specimens greater than 24 hours apart and within 14 days of the onset of paralysis, from at least 80% of AFP cases; and

3) submit all specimens for processing to a WHO accredited laboratory [6].

AFP is a clinical manifestation of many conditions, including poliomyelitis. The WHO have described AFP as a “syndrome characterised by rapid onset of weakness of an individual’s extremities, often including weakness of the muscles of respiration and swallowing, progressing to maximum severity within 1–10 days. The term ‘flaccid’ indicates the absence of spasticity or other signs of central nervous motor system involvement, including hyperflexia, clonus, or extensor plantar responses [7]”. It is imperative to exclude poliovirus as the causative agent of the paralysis.

Each reported case of AFP is reviewed and classified by the national Polio Expert Panel (PEP). The most common cause of non-polio AFP between 1995 and 1999 in Australia was Guillain-Barré syndrome (GBS) [8]. In the same period, 137/143 (96%) of the non-polio AFP cases were hospitalised.

*Whitfield* and *Kelly* conducted a two source capture recapture analysis to estimate the incidence of AFP in an Australian jurisdiction (Victoria) for the years 1998–2000 using the national AFP surveillance system, as used in this current study, and hospital discharge records [9]. They concluded that AFP surveillance in that jurisdiction was insensitive with only 35% of the total number of cases identified reported to the national scheme. From late 2007, the AFP surveillance scheme was complemented by hospital based nurses at four major tertiary paediatric hospitals identifying AFP cases and the target non-polio AFP detection rate has been achieved on nine occasions in Australia since it was established in 1995 and consecutively from 2008 to 2012. Adequate stool specimen collection has never been accomplished in Australia, with collection occurring from approximately 30% of AFP cases reported each year *(Hobday LK, unpublished data).* This sub-optimal performance in virological examination of stool specimens from AFP cases potentially compromises the sensitivity of Australia’s surveillance to detect an imported case of polio and to exclude poliovirus infection [10].

Data from the World Health Organization Western Pacific Region (WPR) for the years 2005 to 2008 inclusive show that the region had variable success in reaching the WHO surveillance targets for non-polio AFP rate and stool collection criteria. On average for the entire Western Pacific region, 74% of the countries satisfied the non-polio AFP notification criteria on a year to year basis while 41% of the countries achieved the stool collection criteria [11]. For example, publications from Hong Kong and Malaysia, within the WPR, and the Netherlands and Italy reported more success achieving the non-polio AFP notification target but were inconsistent in meeting the stool collection criteria [12-15]. Therefore AFP cases are being ascertained worldwide by the surveillance systems but virological testing to exclude poliovirus infection is challenged. For this reason, not all countries have adopted AFP surveillance; Finland chose to employ enterovirus and environmental surveillance in preference to AFP surveillance due to inadequacies and lack of sensitivity using a clinical surveillance scheme [16].

Given the acute and dramatic nature of children presenting with AFP, a hypothesis of this study was that a high proportion of AFP cases would be admitted to an intensive care unit (ICU) due to concerns of progressive paralysis, including respiratory paralysis, and the need for specialised care. Additionally the project aimed to investigate whether the Australian and New Zealand Paediatric Intensive Care (ANZPIC) Registry could identify AFP cases admitted to ICUs but which were not notified through the national AFP surveillance system. The ANZPIC Registry was established in 1997 [17]. Participating ICUs submit case records of an agreed dataset for all children admitted to an ICU in their institution. The system includes codes to classify the reason for admission and associated diagnoses. Could ICUs through the ANZPIC Registry, provide a further opportunity to identify unreported AFP cases and thus enhance AFP surveillance in Australia, consequently improving our ability to achieve the recommended WHO AFP surveillance indicators? To our knowledge this is the first study to investigate the integrity of an intensive care registry to identify AFP cases.

## Methods

### Generation of the AFP and ANZPIC datasets

The data for the linkage study originated from two sources; the national AFP database held at the Victorian Infectious Diseases Reference Laboratory and the ANZPIC Registry held by the Australian and New Zealand Intensive Care Society. AFP notifications are timely, usually within one month of the onset of paralysis, while ANZPIC data is currently submitted to the Registry biannually [18]. For the purposes of this study, the diagnosis assigned to the case by the PEP was accepted as the definitive diagnosis.

### Australian AFP surveillance

AFP surveillance was initiated in 1995 by the Australian Government in collaboration with the Australian Paediatric Surveillance Unit (APSU) in response to the World Health Organization’s recommendation to conduct AFP surveillance as part of the global polio eradication initiative [19]. From 2000, AFP surveillance has been co-ordinated by the National Enterovirus Reference Laboratory at the Victorian Infectious Diseases Reference Laboratory in Melbourne, Australia, in association with the APSU. The APSU facilitates active surveillance of uncommon childhood diseases. Paediatricians return a monthly report card to the APSU indicating if they have diagnosed a case of AFP or other specified clinical presentations. The APSU shares the notification of all AFP cases with the national AFP surveillance co-ordinator who requests the treating doctor to complete a clinical questionnaire that enables the PEP to review the case based on the outcomes of clinical and virological investigations. An additional source of AFP notifications is through Paediatric Active Enhanced Disease Surveillance (PAEDS), which was established in tertiary paediatric hospitals in four states of Australia (New South Wales, South Australia, Victoria and Western Australia) during September 2007 [20]. PAEDS nurses identify AFP cases and report them directly to the national AFP surveillance co-ordinator for review by the PEP. A third source of AFP surveillance is direct notification of cases to the National Enterovirus Reference Laboratory. The final AFP case classifications, epidemiological data and laboratory results are reported to the WHO for inclusion in the Western Pacific Weekly Epidemiological Report.

### AFP dataset

Cases of non-polio AFP between 2005 and 2008 were included in the AFP dataset. The initial dataset comprised 188 cases but exclusions were imposed for patients who were 15 years of age or older (n=18) and non-Australian residents (n=5). One hundred and sixty-five unique AFP cases involving a child less than 15 years of age were identified.

### ANZPIC registry

During the study period, up to 23 Australian and New Zealand ICUs contributed to the ANZPIC Registry including all eight specialist paediatric intensive care units (PICUs). PICUs submit data for all admissions, and other contributing ICUs submit data for all admissions for patients less than 16 years of age. During the period of the study, it is estimated that admissions submitted to the ANZPIC Registry comprised 94% of paediatric admissions to all Australian and New Zealand ICUs [21]. The aims of the uniform system of reporting are to:

•describe paediatric intensive care practices and outcomes in Australia and New Zealand;

•provide contributing units with efficacy and efficiency reports that compare their performance against national and international standards; and facilitate research in paediatric intensive care [16].

### ANZPIC dataset

The ANZPIC Registry uses ‘diagnostic codes’ grouped according to designated major categories to record the clinical diagnosis of children admitted to intensive care. Cases entered into the ANZPIC Registry are assigned one or more diagnoses, as appropriate. Codes for specific microbiologic organisms (such as enterovirus) are assigned based on confirmed laboratory results. Selection of ANZPIC data was performed by filtering for case records submitted to the registry using eight specific diagnostic codes considered compatible with acute neurological presentation with limb weakness. The diagnostic codes selected were: GBS, botulism, myasthenia gravis, spinal cord lesion, myopathy, neuropathy, enterovirus and neurological-other. The designated cases were checked for multiple ICU admissions using the ANZPIC identifiers of date of birth, residential postcode and hospital, with only the initial admission included in the study. Patients aged 15 years or older and patients admitted to ICUs in New Zealand were excluded. There were 880 unique ICU admission records identified in the ANZPIC Registry with acute neurological diagnoses. While cases from ANZPIC originated from both general ICUs and specialist PICUs, 92% of cases analysed in this study originated from the latter.

### Data-linkage

The characteristics of both datasets were examined for gender, age distribution, year of onset/admission and postcode. The proportion of females was 50% in the AFP dataset and 44% in the ANZPIC dataset (Table 1). The proportion of cases by age-groups (0–4, 5–9, 10–14 years of age) in the two datasets were; 43%, 27% and 30% for the AFP surveillance notifications and 50%, 24% and 26% for ANZPIC admissions. The annual number of AFP notifications ranged from 30 to 61 (median 37). The peaks of AFP notification in 2006 (42) and 2008 (61) coincided with the years that Australia successfully met the WHO performance target while the number of AFP cases notified decreased to 30 and 32 for 2005 and 2007, respectively. Annual ANZPIC admissions for the selected diagnoses were relatively steady for 2005, 2007 and 2008 with a peak in 2006 of 249 admissions (range: 203–249 admissions, median 214). The linkage of data was performed using the following identifiers: date of birth, gender and postcode.

**Table 1:** Description of AFP and ANZPIC datasets used for data-linkage

	**AFP**	**ANZPIC**
***Unique cases (n)***	165	880
***Gender***		
Female	50%	44%
Male	50%	56%
***Age group (years)***		
0–4	43%	50%
5–9	27%	24%
10–14	30%	26
***AFP notifications versus ANZPIC admissions by year (n)***		
2005	30	215
2006	42	249
2007	32	203
2008	61	213

Forty-five cases were linked between the two datasets. The linked cases were further scrutinised by comparing the date of onset of paralysis to the date of hospital admission. Three cases were excluded as the date of hospital admission was more than four months after the date of onset of paralysis.

### Ethics

Ethics approval for accessing the ANZPIC Registry data and national AFP surveillance data was granted by the Queensland Children’s Health Services District Ethics Committee (Royal Children’s Hospital, Brisbane) and the Ethics Committee within the Australian Government’s Department of Health and Ageing. Both data repositories contained only de-identified data.

## Results

### Linked cases

Forty-two unique cases were linked between the national AFP database and the ANZPIC Registry. This represented 25% of the total AFP cases classified between 2005 and 2008. Of the 42 linked cases, 19 (45%) were classified by the PEP as GBS, six (14%) as transverse myelitis and three (7%) as acute disseminated encephalomyelitis (ADEM), collectively accounting for two thirds of the cases (Table 2). Four cases of ‘spinal cord injury’ were classified by the PEP at the time but do not fulfil the strict interpretation of the WHO AFP case definition. Thirty-eight (90%) of the linked cases originated from a specialist PICU. Adequate stools were collected from eight (42%) of the GBS cases, two (33%) of the transverse myelitis cases but none from the ADEM cases. A further two cases with the diagnoses of spinal cord injury and spinal muscular atrophy also had adequate stools collected. In total, 12 (29%) of the linked cases satisfied the WHO criteria for adequate stool collection.

**Table 2:** Diagnoses of cases linked between the national AFP database and ANZPIC Registry, 2005–2008

**AFP Diagnosis**	**Total**
Guillain-Barré syndrome	19
Transverse myelitis	6
Spinal cord injury^1^	4
Acute disseminated encephalomyelitis	3
Cord compression	2
Infant botulism	2
Spinal muscular atrophy	2
Tick bite paralysis	2
Leigh's encephalopathy	1
Spinal cord lesion (traumatic myelopathy)	1
Total	42

^1^ Four cases of ‘spinal cord injury’ were classified by the Polio Expert Panel but do not fulfil the strict interpretation of the WHO AFP case definition.

### Unlinked cases

Investigation of the diagnostic codes assigned to the unlinked ANZPIC cases identified a further ten cases of GBS that had not been notified to the AFP surveillance co-ordinator. The only myasthenia gravis case notified to the AFP surveillance system was not identified in the ANZPIC registry. Twelve patients diagnosed with myasthenia gravis were admitted to ICUs but it could not be determined with certainty if any represented the first clinical manifestation rather than treatment for an existing condition.

### Enteroviruses

‘Enterovirus’ is an ANZPIC diagnostic code of interest for poliovirus/enterovirus surveillance since poliovirus can cause meningitis and other non-paralytic conditions. For this reason, many countries, including Australia, perform enterovirus surveillance as a supplement to AFP surveillance to monitor their polio-free status. There were 21 cases of the 880 selected from the ANZPIC Registry which were recorded with enterovirus infection. Only one of the 21 cases was reported to the national AFP surveillance system and was classified as transverse myelitis. Four of the remaining 20 unreported enterovirus cases had an admission code of ‘meningitis’ and a further six were either ‘encephalitis’ or ‘encephalopathy’. The remaining enterovirus cases had accompanying codes including ‘seizures’, ‘respiratory failure’, ‘spinal cord lesion’ and ‘myocarditis’.

## Discussion

This study investigated whether AFP cases were likely to be admitted to an ICU or PICU and whether there were cases recorded in the ANZPIC Registry that had not been notified to the national AFP surveillance system. Forty-two unique cases were linked between the databases indicating that 25% of the total AFP cases in the national dataset included in this study were admitted to a general or specialist ICU.

This finding did not confirm the investigators’ hypothesis that a high proportion of AFP cases in children would be admitted to an ICU or specialist PICU.

Ten additional GBS cases that had not been identified by the existing national AFP surveillance system were found. The GBS cases are of special interest since this was the most common diagnosis attributed to non-polio AFP cases in Australia in 1995–1999 [8]. If the ten unlinked cases had been reported to the national AFP surveillance program it would have increased the number of GBS cases detected from 19 to 29 cases. The inclusion of the previously unidentified AFP cases to the data when Australia did not meet the WHO target (2005 and 2007) improved Australia’s surveillance performance. Although the target rate of 1.0 per 100,000 children less than 15 years old was still not achieved, the adjusted rates for 2005 and 2007 were increased from 0.78 to 0.85 and 0.88 to 0.95 respectively. Thus, reporting AFP cases from within ICU settings could improve the national AFP notification rate.

Myasthenia gravis is a rare auto-immune disease that can require on-going medical care. AFP surveillance is focused on patients presenting with syndromic symptoms for the first time and not related to a previously diagnosed condition. Eleven of the 12 patients listed in the ANZPIC registry with myasthenia gravis also had diagnoses indicative of elective treatment or an acute exacerbation of their existing condition. While the remaining patient had myasthenia gravis listed as the only reason for admission, the case was not counted as unlinked with AFP surveillance as it could not be confirmed that the condition was previously undiagnosed from the information in the registry. One case of myasthenia gravis was notified to the AFP surveillance scheme in 2007 even though it was not listed on the clinical questionnaire. As a result of this study the PEP resolved to include ‘myasthenia gravis’ on the questionnaire as a differential diagnosis for causes of AFP.

The medical conditions of children with AFP, and especially those within an ICU, may impact on their ability to produce stool samples but specimens were received from 14 of the 19 linked GBS cases, although specimens from only eight (42%) cases were adequate according to the WHO recommended criteria. The percentage of linked cases with adequate stools collected (29%) was similar to the average of the national AFP data (33%) for the years of the study, 2005 to 2008 *(Hobday LK, unpublished data)*.

A limitation of the study was the inability to directly compare the selected ANZPIC codes with AFP diagnoses assigned by the PEP. GBS is an admission code on the ANZPIC Registry and so could be linked with the AFP database directly. Transverse myelitis and ADEM represent the second and third most common cause of non-polio AFP, respectively, in Australia after GBS. However, analysis for transverse myelitis and ADEM cases was limited due to these specific diagnoses not being nominated as diagnostic codes on the ANZPIC Registry. Two of the six linked transverse myelitis cases had ‘spinal cord lesion’ as an ANZPIC admission code while one of the three ADEM cases notified through the AFP surveillance system was registered as a ‘spinal cord lesion’. Examination of the unlinked ANZPIC data revealed 66 cases which had been admitted to PICU with the code ‘spinal cord lesion’ and it is postulated that a portion of these cases could be transverse myelitis or ADEM cases. Based on the results of this study, transverse myelitis and ADEM were added to the list of ANZPIC Registry diagnostic codes from 2012.

ICU admission records are currently submitted to the ANZPIC Registry biannually. Therefore this process will not enhance real time surveillance for ascertainment of AFP cases. The results of this study, however, provide an opportunity to raise awareness and motivate improved compliance with AFP reporting and stool collection amongst intensive care clinicians. Use of the data linkage methods we describe to periodically audit reporting and stool collection rates for AFP cases admitted to specialist PICUs may serve to maintain awareness and thereby improve Australia’s performance in meeting the WHO surveillance targets.

## Conclusions

The study indicates that the majority of hospitalised children with AFP are not admitted to intensive care units. Nevertheless, the ANZPIC Registry is a valuable source for identifying additional AFP cases which have not been notified to the national AFP surveillance system. This is especially true for GBS. The addition of ‘transverse myelitis’ and ‘acute disseminated encephalomyelitis’ as diagnostic codes will align the ANZPIC Registry with the three most common causes of non-polio AFP reported in Australia; GBS, transverse myelitis and ADEM. The study highlights the need to increase awareness of the AFP surveillance program in ICUs, particularly specialist PICUs. Periodic audit could provide feedback on the effectiveness of surveillance within Australian ICUs.

## Competing interests

The authors declare that they have no competing interest.

## Authors’ contributions

The study was instigated by DD, AS and BT and LH, JA, PM and MC contributed to the study design and analysis. AFP surveillance data was provided by BT and LH and the ANZPIC Registry data by JA. Data linkage and analysis was performed by TA. The paper was drafted by LH and all authors contributed to and approved the final version.

## Pre-publication history

The pre-publication history for this paper can be accessed here:

http://www.biomedcentral.com/1471-2334/13/384/prepub
